# Rapid recurrence of ovarian mixed sex-cord-stromal tumor associated with DICER1 gene mutation: a case analysis and literature review

**DOI:** 10.1097/MS9.0000000000003569

**Published:** 2025-07-14

**Authors:** Fengyi Zhu, Hai Zhou, Weizheng Li

**Affiliations:** YunFu People’s Hospital, Yunfu, China

**Keywords:** juvenile granulosa cell tumor, ovarian mixed sex-cord-stromal tumor, ovary, rapid recurrence

## Abstract

**Introduction and importance:**

Ovarian tumors are relatively rare in children and adolescent females, with mixed sex-cord-stromal tumors being a specific subtype that has a low incidence and is associated with DICER1 gene mutations.

**Case presentation:**

This case report describes a 14-year-old female patient diagnosed with a mixed sex-cord-stromal tumor associated with a DICER1 gene mutation, who had a rapid recurrence. The patient did not receive standardized chemotherapy after the initial surgery, and the tumor recurred within 6 months, leading to a second surgery and chemotherapy. One year of follow-up showed no recurrence.

**Clinical discussion:**

This case emphasizes the complexity and biological behavior of mixed sex-cord-stromal tumors, providing important differential diagnostic insights for clinical practice. Moreover, with only three cases of recurrence reported globally, it is crucial for clinicians to take this into consideration.

**Conclusion:**

The management experience from this case provides important references for future related research and clinical practice.

## Introduction

Ovarian tumors are relatively rare in children and adolescent females, with an incidence range of 3.7%–25.2%^[1]^. These tumors can primarily be categorized into four major types: epithelial tumors, stromal tumors, sex-cord-stromal tumors, and germ cell tumors. Among them, non-epithelial ovarian tumors have a relatively low incidence, accounting for only 3%–8% of ovarian cancers^[2]^. Non-epithelial ovarian tumors mainly include malignant ovarian germ cell tumors and sex-cord-stromal tumors, with incidences of 2.23 cases and 1.85 cases per 100 000 people, respectively^[3–5]^. Notably, mixed sex-cord-stromal tumors are a rare subtype whose histological features combine those of granulosa cell tumors (GCTs) and Sertoli-Leydig cell tumors, typically occurring in adolescent females^[6]^. Relevant literature indicates that the occurrence of such tumors is closely related to DICER1 gene mutations, which may increase susceptibility to various benign and malignant tumors^[7]^.

**HIGHLIGHTS**

- First reported rapid recurrence (6 months) of pediatric DICER1-mutated ovarian mixed sex-cord-stromal tumor post-surgery without adjuvant chemotherapy.
- Highlights the critical role of standardized chemotherapy in preventing early relapse for DICER1-related ovarian tumors.
- Documents one of only three global cases (including this case report) of recurrent mixed sex-cord-stromal tumors, emphasizing clinical vigilance in adolescents.
- DICER1 mutations identified as potential biomarkers for aggressive behavior in rare ovarian stromal neoplasms.
- Provides a management framework for recurrent pediatric ovarian tumors requiring multimodal therapy.

This case report describes a 14-year-old female patient diagnosed with a mixed sex-cord-stromal tumor associated with a DICER1 gene mutation, who experienced recurrence. After not undergoing standardized chemotherapy, the tumor rapidly recurred within 6 months. However, following surgery and chemotherapy after recurrence, there was no evidence of recurrence during the 1-year follow-up. This is a very rare situation, with only a handful of similar cases reported in the literature. The significance of this case lies in providing an important differential diagnostic approach for clinicians, especially when faced with ovarian tumors exhibiting similar clinical presentations. Through an in-depth analysis of this case, we hope to deepen the understanding of mixed sex-cord-stromal tumors and their biological behavior, providing references for future clinical management.

## Case presentation

### Patient information

**Figure 1.**
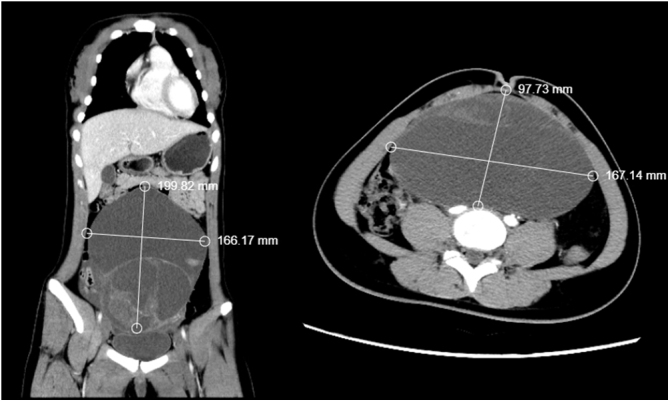
The CT shows a huge cystic-solid mass in the pelvic and abdominal cavity.

**Figure 2.**
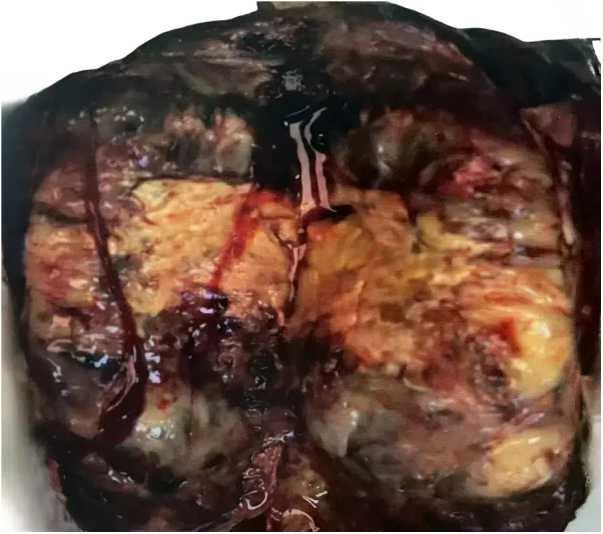
The Sertoli cell component in tumors is arranged in nests or islands, with cells that are round or oval, and the cytoplasm is translucent or mildly eosinophilic.

Patient: Female, 14 years old. A CT scan of the abdomen showed a large cystic solid mass in the abdominal cavity, suspected to originate from a left adnexal lesion, possibly a borderline cystadenoma or teratoma (Fig. 1). Laboratory tests indicated elevated testosterone (TES) at 457.00 ng/ml (normal range: 9.8–82.1), prolactin (PRL) at 40.6 ng/ml, and alpha-fetoprotein (AFP) at 41.0 ng/ml (normal range: 0–10). The patient underwent exploratory laparotomy and excision of the large pelvic tumor. During surgery, the mass was found to contain multiple cystic cavities, with the largest cavity containing light red cystic fluid and a small amount of gelatinous tissue, while the other cavities exhibited bead-like pale yellow gelatinous tissue accompanied by dark red cystic fluid (Fig. 2).

### Pathological examination

**Figure 3.**
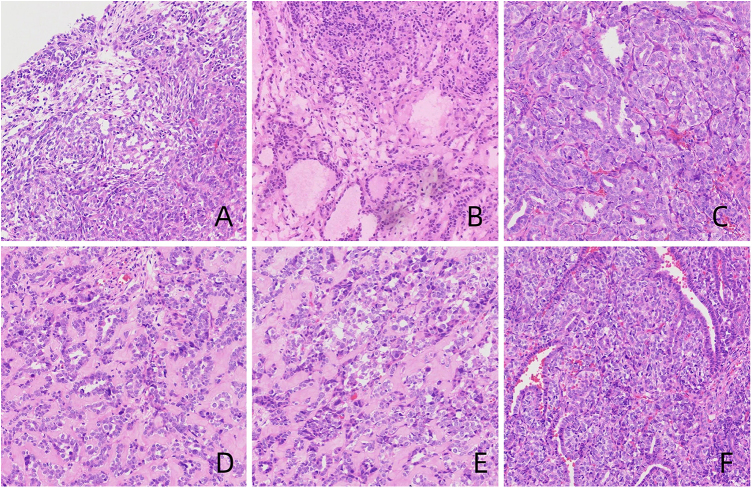
The juvenile granular cell tumor component in the tumor is arranged in a diffuse, sheet-like pattern, with follicle-like cavities visible, containing eosinophilic secretions (A and B). The Sertoli cell component in tumors is arranged in nests or islands, with cells that are round or oval, and the cytoplasm is translucent or mildly eosinophilic (C and D). The tumor shows oval-shaped hollow or solid tubular structures, which are composed of short columnar Sertoli cells, with varying numbers of Leydig cells found between the tubular structures (E and F).

Gross examination revealed a dark red nodular mass measuring approximately 17 × 14 × 4.5 cm, with a cut surface showing a combination of cystic and solid components, and the cysts contained pale yellow gelatinous material; the solid portion was gray-white and dark red, soft in texture, with some areas showing localized bleeding. Microscopic observation showed the tumor was nodular or lobulated, with some areas arranged in tubular, sheet-like, or cord-like patterns of Sertoli cells, abundant eosinophilic cytoplasm, and rare mitotic figures; between the tubular structures, varying numbers of Leydig cells were observed, oval in shape, with abundant eosinophilic cytoplasm, round nuclei, and sparse mitotic figures; some areas of tumor cells were arranged in diffuse sheets, islands, or nests, with round, oval, or polygonal shapes, moderate cytoplasm, clear or slightly eosinophilic, round nuclei, occasional nuclear grooves, clear nucleoli, and visible mitotic figures (Fig. 3).

**Figure 4.**
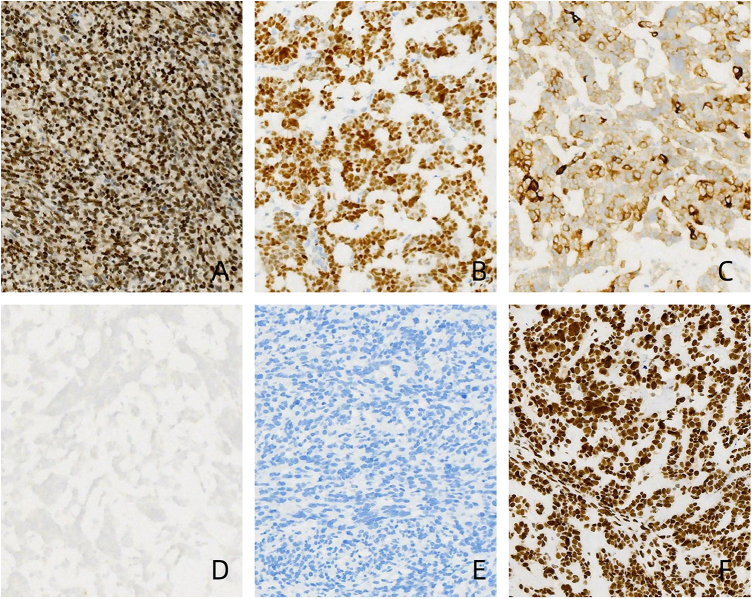
The positive expression image of immunohistochemistry SF-1 (A), WT-1 (B), Inhibin (C), and BRG1 (F) shows positivity. The negative expression images of immunohistochemistry CgA (D) and CR (E) show negativity.

Immunohistochemical results indicated a mixed sex-cord-stromal tumor, accompanied by juvenile-type GCT (40%) and moderately differentiated Sertoli-Leydig cell tumor (60%). The detection results showed: CK (+), PAX-8 (-), WT-1 (+), P53 (+, 60%), NapsinA (-), ER (partial+), PR (few+), Inhibin (partial+), CD99 (-), S-100-), CgA (-), Syn (few+), CD56 (+), CD117 (-), PLAP (-), CD30 (-), BRG-1 (+), Ki67 (+, 40%), CR (-), SF-1 (+) (Fig. 4).

NGS gene testing reported a frameshift mutation in DICER1, Exon16, c.2517delT, p.L840Yfs*6 with an abundance of 39.19%, and a missense mutation in DICER1, Exon25, c.5439G>T, p.E1813D with an abundance of 42.47% (Table 1). This NGS report supports the detection results of mixed sex-cord-stromal tumor.

**Table 1:** Detection results of gene mutations with clear and potential clinical significance: A total of 12 mutations were detected in this test, of which 2 are gene mutations with clear and potential clinical significance. (Class I mutations: mutations with clear clinical significance (A/B level evidence), class II mutations: mutations with potential clinical significance (C/D level evidence), class III mutations: mutations with unclear clinical significance, class IV mutations: mutations with clearly no clinical significance)

Mutation results	Abundance (%)	Mutation level	Clinical significance	Evidence level
DICER1	0.3919	II	Diagnosis	D
Exon16				
c.2517delT p.L840Yfs*6 Codon mutation				
DICER1	0.4247	II	Diagnosis	D
Exon25				
c.5439 G>T p.E1813D Missense mutation				

One month post-surgery, the patient returned for a follow-up, with menstruation restored and cycles approximately normal. Tumor markers such as CA125, human epididymis protein 4, CA153, CA199, CEA, and AFP were all normal. Imaging examinations showed no tumor recurrence. During this period, no standardized chemotherapy was administered. However, a CT follow-up 6 months post-surgery indicated tumor recurrence, with the tumor size measuring 19.8 × 19.1 × 11.0 cm. After surgical treatment, the pathological diagnosis was consistent with the initial diagnosis. The patient underwent a second surgery and received a standardized chemotherapy regimen, including paclitaxel (240 mg) and carboplatin (550 mg), from October 16, 2023, to March 19, 2024, with a total of eight cycles administered^[8]^. One year of follow-up showed no signs of tumor recurrence.

## Discussion

Ovarian mixed sex-cord-stromal tumors exhibit histological features of GCTs and Sertoli-Leydig cell tumors, including poorly differentiated Sertoli cells, Leydig cells, and juvenile GCT tissue^[9]^. According to the WHO fifth edition classification, mixed sex-cord-stromal tumors have been reintroduced, and these two tumor components typically express positive markers for sex-cord-derived tissues (such as inhibin and FOXL2)^[10]^. The vast majority of mixed sex-cord-stromal tumors are benign, and recurrence is relatively rare (WHO fifth edition). Robert Meyer first described this type of tumor in 1930, and subsequently, 28 cases have been reported in the literature^[6,11,12]^. Generally, adult-type GCTs are more common, while mixed sex-cord-stromal tumors with juvenile GCT components are extremely rare, with only six related literature reports^[13]^. Therefore, we share the management experience of this rare mixed sex-cord-stromal tumor case, as it is associated with clinical recurrence and DICER1 mutation, providing some diagnostic insights for clinicians.

### Discussion on DICER1 gene expression in mixed sex-cord-stromal tumors

DICER1 mutations are rare genetic variations that may predispose individuals to various benign and malignant tumors^[13–15]^. In patients carrying DICER1 germline mutations, the onset of gynecological tumors typically occurs from childhood to adulthood^[16–18]^. Pathogenic somatic mutations almost exclusively occur in the form of missense substitutions, affecting five “hotspot” sites in exons 24 and 25 of the RNAse IIIb domain (p.E1705, p.D1709, p.G1809, p.D1810, and p.E1813)^[7,18]^. Ann Marie Mercier revealed the association between mixed sex-cord-stromal tumors (gynandroblastoma) and germline mutations in the DICER1 gene^[9,19]^. Relevant literature indicates that DICER1 mutations are usually associated with mixed sex-cord-stromal tumors, with an incidence range of 19%–80%^[7,19,20]^. However, the NGS gene testing report in this study showed DICER1, Exon16, p.L840Yfs*6 frameshift mutation and Exon25, p.E1813D missense mutation, further validating our diagnosis. There is ongoing debate in the literature regarding whether DICER1 mutations arise due to the presence of SLCT (Sertoli-Leydig cell tumor) components or if these mutations are universally present across various mixed sex-cord-stromal tumor categories^[7]^. SLCT is currently classified into three molecular subtypes with unique clinical pathological features: DICER1 mutant type, FOXL2 mutant type, and DICER1/FOXL2 wild type^[21]^. Most SLCTs are associated with DICER1 gene variations, both germline and somatic changes, with reported frequencies varying, reaching up to 97%^[22–28]^. However, a study by Wang Y indicated that sequencing of 16 mixed sex-cord-stromal tumors suggested that one subgroup of these tumors may originate from SLCT and contain only one morphologically similar component to GCTs^[19]^. A literature review showed that sequencing of 75 JGCT cases confirmed six cases with somatic DICER1 mutations, with an incidence of 8%, and reported two cases possibly related to germline DICER1 syndrome JGCT^[20,24,25,29–31]^. In summary, the DICER1 gene can be expressed in both supportive stromal tumors and GCTs, further validating the diagnosis of mixed sex-cord-stromal tumors. However, the mixed sex-cord-stromal tumor in this case included juvenile GCT and supportive-stromal cell tumor. This study will further explore the relationship between them.

### Juvenile GCT and Sertoli-Leydig cell tumor

Ovarian GCT is a rare ovarian tumor, accounting for approximately 0.6%–3% of all ovarian tumors, derived from ovarian sex-cord cells, and is a low-grade malignant tumor^[32–34]^. It can occur in women from newborns to postmenopausal, with the highest incidence in perimenopausal and postmenopausal women. Based on clinical presentation and pathological histological characteristics, it is divided into adult-type granulosa cell tumor and juvenile-type granulosa cell tumor (JGCT), with the juvenile type being rarer, accounting for only 5% of GCTs, and about 97% of JGCT patients are under 30 years of age^[32,33,35]^. JGCT tumor cells are arranged in diffuse sheets, nests, or follicular patterns, with the common feature of follicular structures being composed of generally medium-sized or smaller irregularly shaped cavities, with eosinophilic or basophilic secretions within the cavities, and cells are round or polygonal, with moderate cytoplasm, clear or slightly eosinophilic, round nuclei, lacking Call-Exner bodies and nuclear grooves, with frequent nuclear atypia and visible mitotic figures (high counts can exceed 5/10 HPF)^[35]^. The immunophenotype of GCTs is CK18 and CD99 positive, with some literature reporting a positivity rate for a-Inhibin of 66%–94%, but recent studies report a positivity rate of 25%^[36]^. Calretinin and SF-1 are commonly positive, while CEA and EMA are negative^[36]^.

Ovarian Sertoli-Leydig cell tumor (SLCT) is a relatively rare sex-cord-stromal tumor and is the most common tumor associated with masculinization, accounting for about 0.5% of all ovarian tumors^[37,38]^. It commonly occurs in women aged 20–30, but can also occur before puberty or after menopause^[38]^. Due to the tumor cells’ ability to secrete androgens, 25%–77% of patients may exhibit defeminization characteristics (such as oligomenorrhea, amenorrhea, breast atrophy, etc.), gradually developing symptoms of masculinization such as deepening voice, hirsutism, acne, and clitoromegaly^[38,39]^. SLCT is composed of Sertoli cells and Leydig cells of varying degrees of differentiation mixed in different proportions^[40]^. Moderately differentiated SLCT tumor cells are nodular or lobulated, separated by loosely edematous fibrous or fibrous mucinous stroma, with Sertoli cells arranged in sheets, cords, or focal patterns, abundant eosinophilic cytoplasm, low columnar shape, and some cells may exhibit nuclear grooves, with minimal cellular atypia and rare mitotic figures; between tubular structures, varying numbers of Leydig cells are observed, appearing in sheets, clusters, or scattered singly, oval in shape, with abundant eosinophilic cytoplasm and small round nuclei. Lipid vacuoles can be seen in the cytoplasm of both Sertoli and Leydig cells, and occasionally bizarre nuclei are observed^[38,41]^. The immunophenotype of this tumor expresses Inhibin, CD99, Calretinin, AE1/AE3, low molecular weight keratin, CD10, Vim, SMA, etc., and rarely expresses EMA^[38]^.

The diagnosis of mixed sex-cord-stromal tumors primarily relies on histological features, with diagnostic criteria requiring that the secondary tumor component accounts for at least 10% of the entire tumor^[42]^. In summary, the immunohistochemical results of this case (CR (few+), SF-1 (+), WT-1 (+)) and the microscopic morphology further validated the diagnosis of GCT and SLCT. Therefore, the diagnosis of mixed sex-cord-stromal tumor can be established.

Currently, the most diagnostically significant aspect of ovarian mixed sex-cord-stromal tumors is genetic testing. This testing identified a total of 12 mutations, of which 2 are clearly related to clinically significant gene testing. However, among the class III gene mutation testing results, the genes with higher abundance mutations include MKNK1, POLE, RET, RTEL1, RUNX1T1, TAP1, indicating that these gene mutations may be related to sex-cord-stromal tumors or require further research to explore unknown tumor subtypes (Table 2). The overexpression of DICER1 may have a certain relationship with RET, which has not been explored in depth in this article.

**Table 2:** Class III gene mutation detection results

Gene	Mutation type	Gene region	Nucleotide change	Amino acid change	Abundance (%)
DAXX	Non-coding deletion mutation	Exon5	c.1353_1355delGGA	p.E457delE	0.033
MKNK1	Missense mutation	Exon3	c.13G>A	p.E5K	0.4267
MSI1	Non-coding deletion mutation	Exon12	c.834_842delGGCAGCGGC	p.A279_A281delAAA	0.0069
PAK1	Missense mutation	Exon6	c.545A>C	p.D182A	0.0025
POLE	Missense mutation	Exon18	c.1988G>T	p.C663F	0.459
RET	Missense mutation	Exon8	c.1618A>G	p.R540G	0.4874
RET	Missense mutation	Exon3	c.431G>A	p.R144H	0.4852
RTEL1	Missense mutation	Exon31	c.3135C>A	p.H1045Q	0.4763
RUNX1T1	Cutting mutation	Intron	c.28+26833G>C	.	0.4541
TAP1	Nonsense mutation	Exon1	c.135_136delinsAT	p.E46*	0.4613

### Discussion on recurrence of mixed sex-cord-stromal tumors

Interestingly, current reports on recurrence cases of mixed sex-cord-stromal tumors are relatively scarce, with only two cases reported to date, the first of which was published in 2007^[9,43]^. This case involves a recurrent mixed sex-cord-stromal tumor associated with juvenile GCT and DICER1 gene mutation, warranting further research and discussion. The mechanisms of occurrence and development of this tumor remain unclear, but it is generally believed to originate from a single progenitor cell that can differentiate into male and female characteristics^[42]^. During embryonic development, the mesoderm of the urogenital ridge forms gonadal tissues, which constitute the endocrine active part of the gonads, including granulosa cells, Sertoli cells, ovarian stromal cells, and Leydig cells^[44]^. According to existing case reports, most such tumors are benign, and stage I tumors can generally be cured after resection^[13]^. Additionally, literature indicates that these patients typically belong to low-grade tumors, and mixed sex-cord-stromal tumors with juvenile GCT components have shown no signs of recurrence during clinical follow-up^[13]^. However, a study by Neubecker RD indicated that cases of mixed sex-cord-stromal tumors with 40% adult-type GCTs experienced tumor recurrence^[44]^. Therefore, we must question whether the reason for recurrence is related to the proportion of GCTs. The malignancy of GCTs is generally higher than that of other sex-cord-stromal tumors, and juvenile GCTs have a higher malignancy than adult types. However, a study by Adam Wilberger showed that mixed sex-cord-stromal tumors with 45% juvenile GCTs, despite having mitotic figures around 3–6/10 HPF, did not experience tumor recurrence^[6]^. Although the mixed sex-cord-stromal tumor in this case also had a high proportion of juvenile GCTs (40%), we still lack sufficient evidence to prove that tumor recurrence is associated with the proportion of GCTs. Furthermore, we speculate whether recurrence is related to the degree of differentiation of SLCT components. However, in Adam Wilberger’s study, the degree of differentiation of SLCT components was moderate, and no tumor recurrence was observed, while the mixed sex-cord-stromal tumor in this case also contained moderately differentiated SLCT components but experienced tumor recurrence^[6]^. Therefore, we need to consider whether factors such as the lack of standardized treatment led to recurrence. However, post-operative tumor markers and imaging examinations indicated that the tumor had been completely removed. Due to the rarity of this case, research is challenging, and further exploration of the reasons for recurrence is urgently needed. Neubecker RD believes that recurrent tumors do not necessarily imply dedifferentiation or malignant transformation, as this is often accompanied by broader molecular mutation events^[44]^. Whether tumor recurrence is related to mutations in MKNK1, POLE, RET, RTEL1, RUNX1T1, TAP1 still requires further research for validation.

### On the necessity of standardized postoperative chemotherapy

According to previous case reports, most tumors are benign, and cases staged as stage I can generally be cured after resection^[45]^. However, this case experienced tumor recurrence 6 months after surgery without receiving standardized postoperative treatment. The patient underwent a second surgery and received a standardized chemotherapy regimen, including paclitaxel (240 mg) and carboplatin (550 mg), from October 16, 2023, to March 19, 2024, with a total of 8 cycles administered^[8]^. One year of follow-up showed no signs of tumor recurrence. For this patient, the time from initial onset to recurrence was extremely short, only 6 months, which significantly differs from the descriptions of recurrence in existing literature^[6,43]^. Additionally, the maximum diameter of the tumor at initial diagnosis was approximately 17 cm, while at recurrence, it had increased to approximately 20 cm, indicating a higher degree of malignancy and rapid recurrence, which is notably different from reported mixed sex-cord-stromal tumors, necessitating greater attention and follow-up. After tumor recurrence, we administered standardized chemotherapy, and currently, the patient’s testosterone and tumor markers (such as CA125, human epididymis protein 4, CA153, CA199, CEA, AFP) are all normal, with imaging examinations also showing no tumor recurrence. Therefore, we need to reconsider that not all mixed sex-cord-stromal tumors can achieve cure after resection. However, regarding why this patient had such a high degree of malignancy and rapid recurrence, we have not explored in depth, and we look forward to more laboratory studies providing relevant evidence in the future. In summary, the diagnosis, treatment, and follow-up of this tumor differ from previous cases, and we need to be more careful.

### Research limitations

(1) Sample scarcity limitation: This study is a single-center case report, limited by the extreme rarity of ovarian mixed germ cell tumors (only three cases of recurrence reported globally), making it difficult to establish a statistically significant prognostic model from a single case. (2) Follow-up time limitation: Although a 1-year disease-free survival was achieved after secondary treatment, DICER1-related tumors may have a risk of late recurrence (with literature reporting up to 10 years), necessitating an extended follow-up period to verify long-term efficacy. (3) Molecular mechanisms not fully elucidated: Although DICER1 hotspot mutations were detected, whole-exome sequencing or single-cell sequencing was not conducted, making it impossible to rule out the synergistic effects of other genes such as RET and POLE, which may affect the understanding of tumor heterogeneity and recurrence-driving factors.

### Clinical practice recommendations

1. Standardized diagnostic process:
  - It is recommended that all patients with mixed germ cell tumors undergo mandatory DICER1 gene testing (covering at least Exon 16, Exon 24–25 hotspot regions).

2. Treatment decision framework:
  - Even if R0 resection is achieved during the initial surgery, for patients with >30% JGCT components or those with DICER1 biallelic mutations, it is recommended to refer to the ovarian malignant germ cell tumor regimen (such as BEP or paclitaxel/carboplatin) for adjuvant chemotherapy.
  - Establish a multidisciplinary team to assess the balance between endocrine abnormalities (such as testosterone level monitoring) and chemotherapy tolerance.

3. Data sharing mechanism:
  - Advocate for the establishment of an international registry for rare germ cell tumors (such as connecting to the DICER1 syndrome consortium) to standardize the recording of tumor component ratios, mutation profiles, and chemotherapy response data.
  - Encourage the development of organoid models for in vitro validation of chemotherapy drug sensitivity (especially for recurrent cases).

## Data Availability

All data generated or analyzed during this study are included in this published article.
